# A novel tumor spheroid model identifies selective enhancement of radiation by an inhibitor of oxidative phosphorylation

**DOI:** 10.18632/oncotarget.27166

**Published:** 2019-09-03

**Authors:** Henning Karlsson, Wojciech Senkowski, Mårten Fryknäs, Sharmineh Mansoori, Stig Linder, Joachim Gullbo, Rolf Larsson, Peter Nygren

**Affiliations:** ^1^Department of Medical Sciences, Uppsala University, Uppsala, Sweden; ^2^Department of Medical and Health Sciences, Linköping University, Linköping, Sweden; ^3^Cancer Center Karolinska, Department of Oncology and Pathology, Karolinska Institute, Stockholm, Sweden; ^4^Department of Immunology, Genetics and Pathology, Uppsala University, Uppsala, Sweden

**Keywords:** spheroid, tumor model, high-throughput, radiosensitizer, hypoxia

## Abstract

There is a need for preclinical models that can enable identification of novel radiosensitizing drugs in clinically relevant high-throughput experiments. We used a new high-throughput compatible total cell kill spheroid assay to study the interaction between drugs and radiation in order to identify compounds with radiosensitizing activity. Experimental drugs were compared to known radiosensitizers and cytotoxic drugs clinically used in combination with radiotherapy. VLX600, a novel iron-chelating inhibitor of oxidative phosphorylation, potentiated the effect of radiation in tumor spheroids in a synergistic manner. This effect was specific to spheroids and not observed in monolayer cell cultures. In conclusion, the total cell kill spheroid assay is a feasible high-throughput method in the search for novel radiosensitizers. VLX600 shows encouraging characteristics for development as a novel radiosensitizer.

## INTRODUCTION

Radiotherapy is a cornerstone in cancer treatment and is often combined with cytotoxic drugs with the aim to enhance the antitumor effects. One example is 5-fluorouracil (5-FU)-based chemoradiotherapy that is a standard of care for locally advanced rectal cancer [1]. Other drugs used for the purpose of sensitization to radiation are the platinums, taxanes [2] and mitomycin for the treatment of, *e.g.,* cancer of the lung, esophagus and anus [2–4]. Some new targeted drugs have also been successfully combined with radiotherapy [5].

However, although combined treatment may provide improved tumor control, enhanced normal tissue toxicity is often also observed. The common view that the cytotoxic drugs result in an improved therapeutic ratio, compared with radiotherapy only, has recently been questioned [6–10]. Thus, a drug that more selectively sensitizes cancer cells to radiation would be of substantial value in radiotherapy and allow for a lower radiation dose to be effective against malignant cells while sparing neighboring normal cells or, alternatively, to increase the radiation dose to achieve better tumor control at similar normal tissue toxicity.

It is well established that hypoxic areas in tumors are associated with radiation resistance [11]. A major cause for radiation resistance is that lack of oxygen reduces the number of DNA double-strand breaks (DSBs) caused by radiation induced formation of free oxygen radicals. However, currently there is a lack of established strategies to reduce tumor hypoxia in order to selectively sensitize tumors to radiotherapy [11, 12].

Hypoxia induce stem cell-like properties in cancer cells which can also contribute to chemoresistance [13, 14]. Although such cancer stem-like cancer cells may constitute less than a few per cent of the tumor mass, they are thought to be responsible not only for resistance to therapy but also for cancer recurrence [13, 14].

In colorectal cancer, microenvironmental factors that maintain the pool of intestinal stem cells also provide the conditions necessary for proliferation of cancer stem-like cells [13]. Since hypoxia not only is the most important microenvironmental driving force for angiogenesis but can induce both resistance to therapy and increase the metastatic potential of colorectal cancer cells, it would be of considerable value to find a drug that enables reversal of hypoxia and selective radiosensitization of hypoxic cancer stem-like cells [13, 15]. The inhibition of oxidative phosphorylation in human cancer cells, *e.g.,* colon cancer, under hypoxic conditions has been shown to be a promising strategy for anticancer treatment [16–18].

One major problem in the search for novel radiosensitizers is to study the interplay between drugs and radiation in clinically relevant high-throughput models. Therefore, a relevant high-throughput preclinical model that could identify synergistic effects between drugs and radiation would be of substantial value.

In this study, a new high-throughput compatible tumor spheroid model was used to study the interaction between drugs and radiation in order to identify drugs with putative beneficial interaction patterns, *i.e.,* drugs that potentiate the effect of radiation in a synergistic manner. Spheroid models with the HCT116 colon cancer cell line have been found robust and replicative and have also been useful in screening for compounds that reduce oxygen consumption rate in colon cancer cells both *in vitro* and *in vivo* [16, 19]. In the spheroid model used in this study, we found that VLX600, a novel iron-chelating inhibitor of oxidative phosphorylation that has previously been shown to reverse hypoxia in HCT116 spheroids [16, 17], selectively enhanced radiation sensitivity of tumor cells grown as spheroids. VLX600 is suggested to be a candidate for further development into a drug for combination with radiotherapy.

## RESULTS

### Spheroid experiments

#### Spheroid morphology and effect of radiation

**Figure 1:**
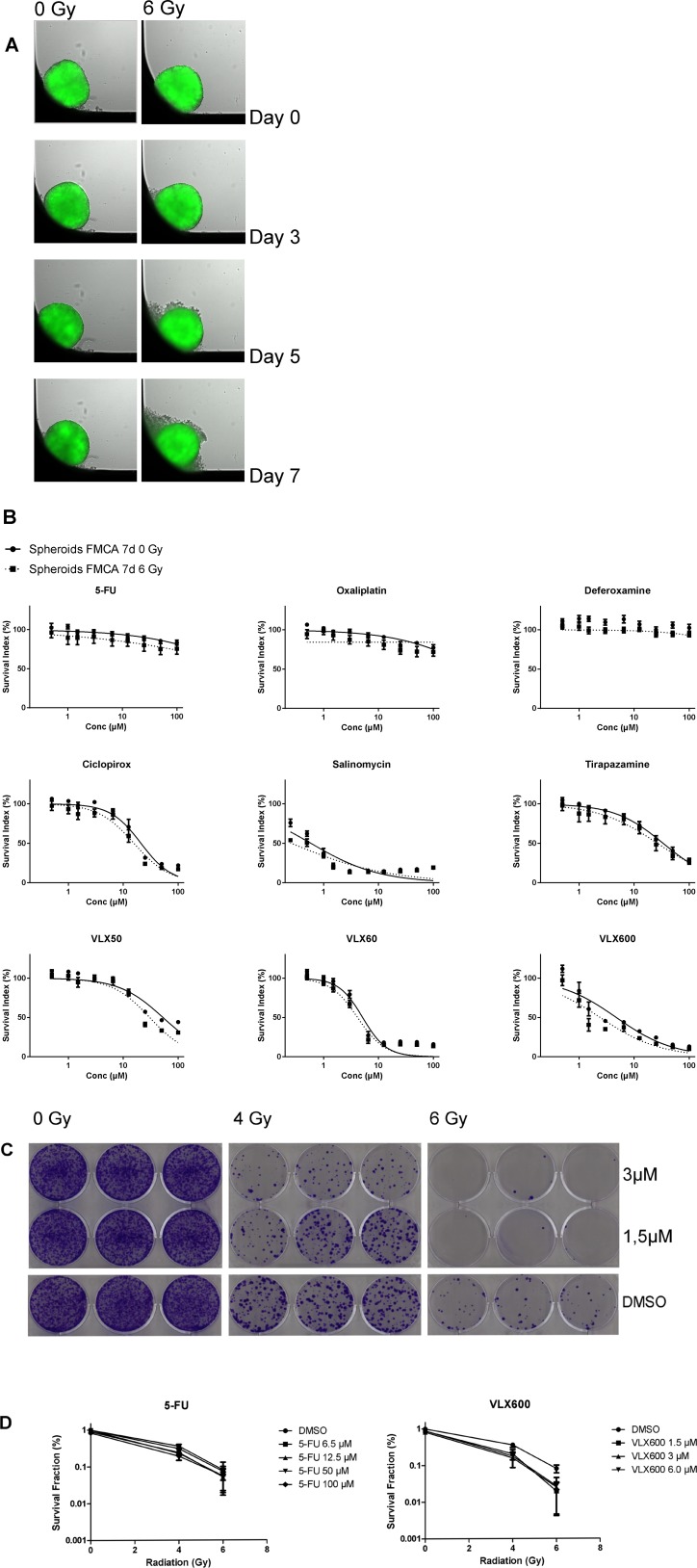
**(A)** HCT116 GFP cells cultured as spheroids for 7 days, irradiated day 0 and then analyzed. Control spheroids (left column) vs irradiated spheroids (right column). The spheroids were typically 400–500 μm in diameter at day 0. (**B**) Cell survival in the FMCA assay, expressed as SI of HCT116 GFP cells cultured as spheroids for 7 days, then incubated with drugs for 7 days with irradiation (6 Gy) at 4–6 h after addition of drug. Mean ± SEM based on 3–7 independent experiments, with duplicate wells for each drug concentration. (**C**) Clonogenic assay with VLX600, shown as growth of HCT116 GFP cells cultured as spheroids for 7 days, then irradiated (6 Gy) 4–6 h after drug addition and 20 h later dissociated into single cells, transferred to 6-well plates and incubated for 10 days. Triplicate wells for each drug concentration. (**D**) Cell survival in the clonogenic assay, expressed as survival fraction of HCT116 GFP cells cultured as spheroids for 7 days, then irradiated (6 Gy) 4–6 h after drug addition and 20 h later dissociated into single cells, transferred to 6-well plates and incubated for 10 days. Mean ± SEM based on 2–3 independent experiments, with triplicate wells for each drug concentration.

Homogenous and equally sized spheroids were formed as described below and shown in Figure 1A. Whereas control spheroids were visually unaffected during the 7 days, irradiated spheroids turned slightly dissociated during the same time period (Figure 1A).

#### Total cell kill assay

Radiation had very little effect on cell survival in spheroids as measured in the fluorometric microculture cytotoxicity assay (FMCA) (SI 93.1 ± 4.19% (mean ± SEM) at 6 Gray (Gy) (Figure 1B) which was in accordance with results from the green fluorescent protein (GFP) assay (not shown). Whereas spheroids were very resistant to 5-FU, oxaliplatin and deferoxamine alone, other drugs tested were more active (Figure 1B). VLX600 was the only drug that showed consistent and significant synergistic effects with radiation in both the FMCA and the GFP assay and this interaction was seen at relatively low concentrations (1–6.5 μM; Figure 1B and detailed in Tables 1–2).

**Table 1:** Interaction ratios in the FMCA assay of drug and radiation combinations in HCT116 GFP cells cultured as spheroids for 7 days, then incubated with drugs for 7 days with irradiation (6 Gy) at 4–6 h after addition of drug

**SI_o_/SI_e_**	**5-FU**	**Oxaliplatin**	**Deferoxamine**	**Ciclopirox**	**Salinomycin**	**Tirapazamine**	**VLX50**	**VLX60**	**VLX600**
Highest conc.	**0.96**	1.00	**0.99**	N/A	N/A	1.09	**0.78**	N/A	N/A
	**0.93**	**0.92**	**0.97**	N/A	N/A	**0.91**	**0.80**	N/A	N/A
	**0.95**	**0.99**	1.02	**0.85**	N/A	**0.94**	**0.79**	N/A	N/A
	1.03	**0.95**	1.02	**0.92**	N/A	1.04	1.06	N/A	**0.81**
	**0.97**	**0.98**	**0.95**	1.04	N/A	1.04	1.03	**0.96**	**0.92**
	**0.99**	**0.96**	**0.98**	0.94^*^	N/A	**0.98**	1.06	**0.93**	0.84^*^
	**0.98**	1.01	**0.94**	**0.96**	1.19	**0.99**	**0.95**	**0.98**	0.72^*^
	**0.92**	1.04	**0.98**	**0.98**	0.87^*^	**0.94**	1.02	1.01	**0.95**
Lowest conc.	1.02	0.95^*^	1.02	1.00	**0.87**	1.02	1.09	1.01	**0.94**

One-sample *t*-test. Mean interaction ratios (SI_o_/SI_e_) significantly different than 1.0 (two-tailed *p*-value <0.05) are shown with an asterisk. Mean interaction ratios (SI_o_/SI_e_) less than 1 indicates synergy and are shown in bold. Drug concentrations for all drugs except salinomycin ranged 100–0.5 μM with 2-fold dilution steps. Drug concentrations for salinomycin ranged 50–0.25 μM. Mean values are based on 3–7 independent experiments, with duplicate wells for each drug concentration. N/A, SI_d_ ≤25%.

**Table 2:** Interaction ratios in the GFP assay of drug and radiation combinations in HCT116 GFP cells cultured as spheroids for 7 days then incubated with drugs for 7 days with irradiation (6 Gy) at 4–6 h after addition of drug

**SI_o_/SI_e_**	**5-FU**	**Oxaliplatin**	**Deferoxamine**	**Ciclopirox**	**Salinomycin**	**Tirapazamine**	**VLX50**	**VLX60**	**VLX600**
Highest conc.	**0.97**	1.02	**0.96**	N/A	N/A	1.09	**0.92**	N/A	1.05
	1.01	**0.98**	1.02	N/A	N/A	1.04	0.84^*^	N/A	1.00
	**0.97**	1.02	**0.94**	N/A	N/A	1.09	0.89^*^	N/A	**0.94**
	1.02	1.00	**0.99**	1.01	N/A	1.03	1.04	N/A	0.89^*^
	**0.98**	**0.99**	1.01	1.02	N/A	1.02	**0.95**	1.05	0.85^*^
	**0.99**	**0.97**	1.01	1.00	N/A	1.08^*^	**0.98**	1.02	0.85^*^
	1.01	**0.99**	**0.98**	1.02	1.33	1.03	1.00	**0.98**	0.86^*^
	1.01	1.01	1.00	**0.98**	1.04	1.04	**0.98**	**0.99**	**0.93**
Lowest conc.	1.04	**0.99**	1.02	1.01	1.08	1.01	1.00	0.94^*^	1.02

One-sample *t*-test. Mean interaction ratios (SI_o_/SI_e_) significantly different than 1.0 (two-tailed *p*-value <0.05) are shown with an asterisk. Mean interaction ratios (SI_o_/SI_e_) less than 1 indicates synergy and are shown in bold. Mean values are based on 3–8 independent experiments, with duplicate wells for each drug concentration. For drug concentrations, see Table 1. N/A, SI_d_ ≤25%.

#### Clonogenic assay

The synergistic effect between VLX600 and radiation in spheroids indicated by measurement of cell viability in the total cell population was further investigated in a clonogenic assay (Figure 1C and 1D). Whereas the clonogenicity of cells in control spheroids was clearly affected by radiation only (SF 36.1 ± 5.16% and 8.13 ± 1.94% (mean ± SEM) at 4 and 6 Gy, respectively, Figure 1D), VLX600 at low concentrations (1.5–6.5 μM) synergistically sensitized the cells to radiation in a radiation dose-dependent manner (Figure 1C and 1D, Table 3). Although 5-FU at high concentrations (50–100 μM) showed radiosensitizing effects, the effect was not as strong as with VLX600 and was not radiation dose-dependent (Figure 1D and Table 3).

**Table 3:** Interaction ratios in the clonogenic assay of drug and radiation combinations in HCT116 GFP cells cultured as spheroids for 7 days, then irradiated (6 Gy) 4–6 h after drug addition and 20 h later dissociated into single cells, transferred to 6-well plates and incubated for 10 days

**SI_o_/SI_e_**	**5-FU + 4 Gy**	**5-FU + 6 Gy**	**VLX600 + 4 Gy**	**VLX600 + 6 Gy**
Highest conc.	0.57^*^	**0.67**	0.51^*^	0.30^*^
	0.61^*^	**0.58**	**0.57**	0.36^*^
	N/A	N/A	**0.78**	0.29^*^
	**0.88**	**0.96**	-	-
Lowest conc.	**0.81**	**0.90**	-	-

One-sample *t*-test. Mean interaction ratios (SI_o_/SI_e_) significantly different than 1.0 (two-tailed *p*-value <0.05) are shown with an asterisk. Mean interaction ratios (SI_o_/SI_e_) less than 1 indicates synergy and are shown in bold. Drug concentrations for 5-FU ranged 100–6.5 μM and for VLX600 6.5–1.5 μM with 2-fold dilution steps. Mean values are based on 2–3 independent experiments, with triplicate wells for each drug concentration. N/A, 5-FU at 25 μM not shown due to technical failure. -, not tested.

### Monolayer experiments

#### Total cell kill assay

**Figure 2:**
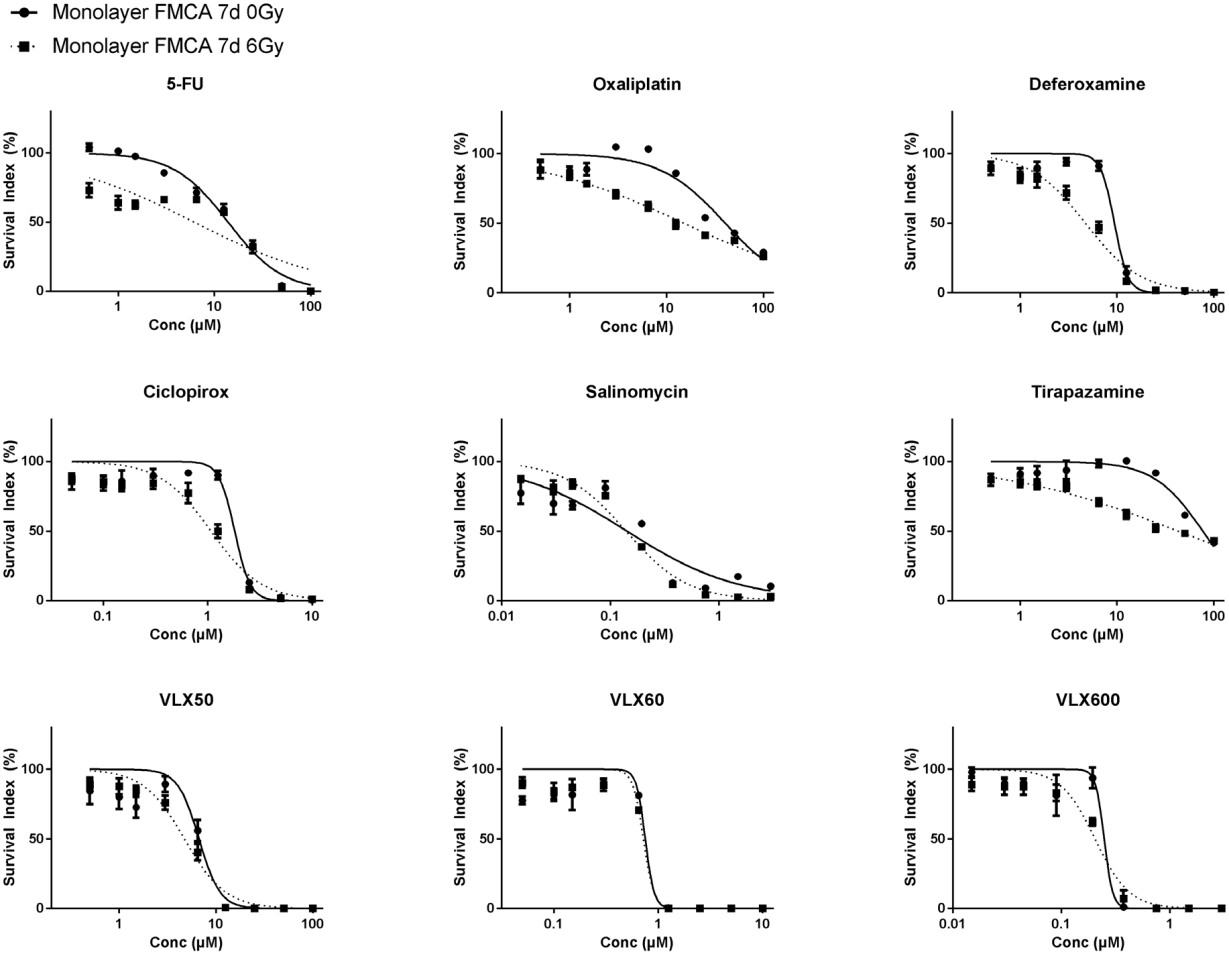
Cell survival in the FMCA assay, expressed as SI of HCT116 GFP cells cultured as monolayers and incubated with drugs for 7 days with irradiation (6 Gy) at 4–6 h after addition of drug. Mean ± SEM based on 3 independent experiments, with duplicate wells for each drug concentration.

As expected, monolayer cells, compared to cells grown as spheroids, were more sensitive to both radiation (SI 74.3 ± 5.93%; mean ± SEM) at 6 Gy) and all drugs alone (Figure 2). Although several drugs showed trends toward synergistic effects with radiation, none showed consistent statistically significant synergy with radiation in the FMCA or the GFP assay and VLX600 was one of the least effective radiosensitizing drugs in these experiments (Table 4 for FMCA and not shown for the GFP assay, respectively).

**Table 4:** Interaction ratios in the FMCA assay of drug and radiation combinations in HCT116 GFP cells cultured as monolayers and incubated with drugs for 7 days with irradiation (6 Gy) at 4–6 h after addition of drug

**SI_o_/SI_e_**	**5-FU**	**Oxaliplatin**	**Deferoxamine**	**Ciclopirox**	**Salinomycin**	**Tirapazamine**	**VLX50**	**VLX60**	**VLX600**
Highest conc.	N/A	1.21^*^	N/A	N/A	N/A	1.40^*^	N/A	N/A	N/A
	N/A	1.19	N/A	N/A	N/A	1.08	N/A	N/A	N/A
	1.37	1.03	N/A	N/A	N/A	0.77^*^	N/A	N/A	N/A
	1.32	0.77^*^	N/A	0.74^*^	N/A	**0.83**	N/A	N/A	N/A
	1.28	**0.81**	**0.70**	1.13^*^	**0.96**	**0.98**	1.02	1.19	0.91
	1.05	**0.91**	1.03	1.28	1.28	1.21	1.16	1.34	1.49
	**0.87**	1.22	1.24	1.35	1.67^*^	1.24	1.65	1.47^*^	1.31
	0.85^*^	1.31	1.36	1.38	1.63	1.28	1.51	1.41	1.32
Lowest conc.	**0.95**	1.36	1.33^*^	1.40	1.58	1.34	1.46	1.58^*^	1.23

One-sample *t*-test. Mean interaction ratios (SI_o_/SI_e_) significantly different than 1.0 (two-tailed *p*-value <0.05) are shown with an asterisk. Mean interaction ratios (SI_o_/SI_e_) less than 1 indicates synergy and are shown in bold. Drug concentrations for 5-FU, oxaliplatin, deferoxamine, tirapazamine and VLX50 ranged 100–0.5 μM, for salinomycin and VLX600 3–0.015 μM and for ciclopirox and VLX60 10–0.05 μM with 2-fold dilution steps. Mean values are based on 3 independent experiments, with duplicate wells for each drug concentration. N/A, SI_d_ ≤25%.

#### Clonogenic assay

Compared to cells from spheroids, monolayer cells were more sensitive to radiation as well as to VLX600 and 5-FU in the clonogenic assay. VLX600 and 5-FU were tested at concentrations close to their IC_50_-values in the FMCA and the GFP assay (0.3 μM and 25 μM, respectively). SF was less than 10% for both drugs at these concentrations and SF after 4 Gy was 10%, to be compared with 36% after 4 Gy in spheroid experiments. No statistically significant synergistic effects between radiation and the two drugs were seen (not shown). However, the SF for drug only was too low to reliably assess synergy in this assay.

#### Immunohistochemistry (IHC) for assessment of DNA double-strand breaks

**Figure 3:**
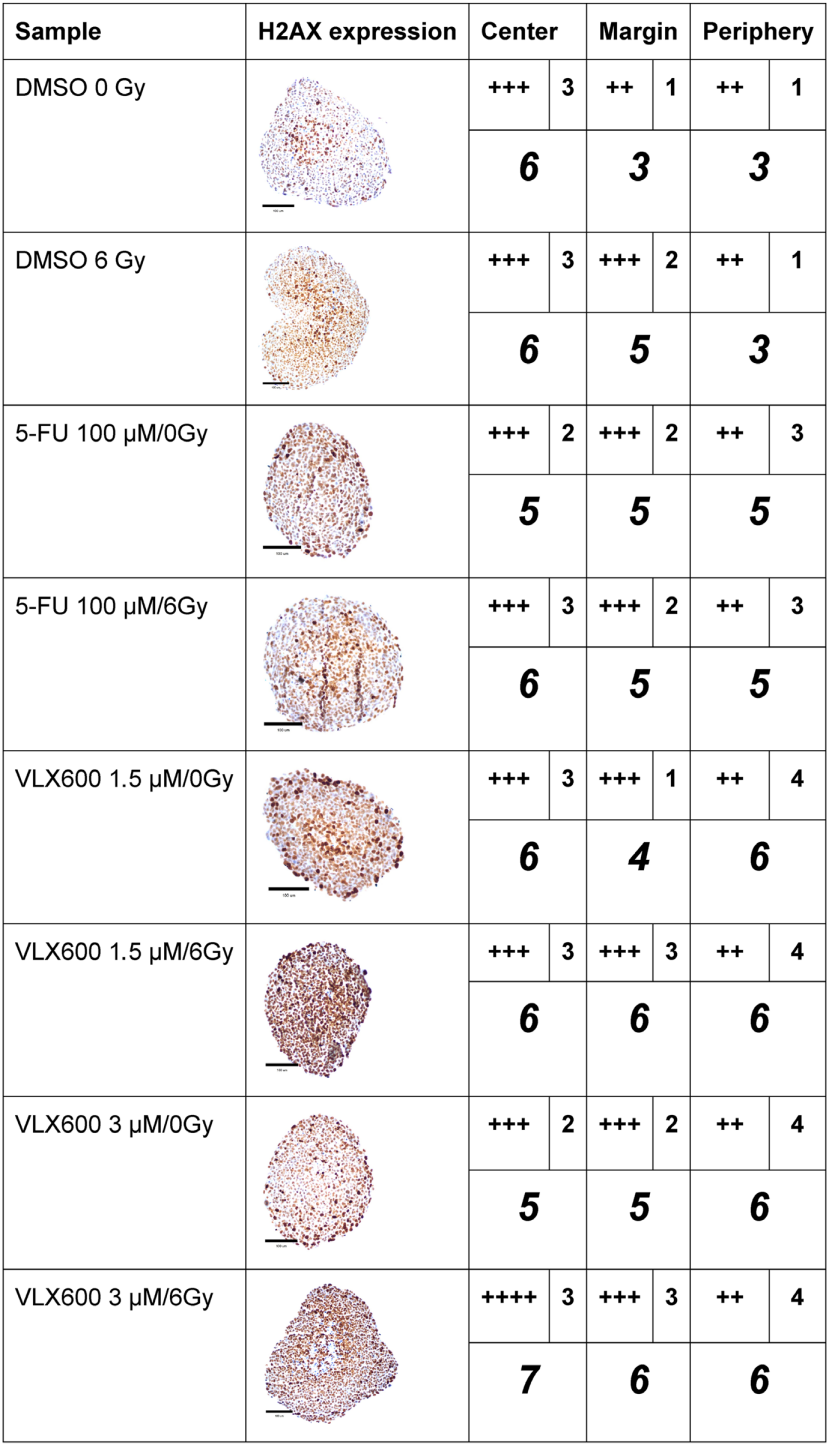
Immunohistochemical expression (centrally and peripherally in spheroids) of gamma-H2AX in HCT116 GFP cells cultured as spheroids for 7 days, then irradiated (6 Gy) at 4–6 h after addition of drug and harvested 24 h later. A typical expression pattern from each sample is shown in the second column. A light microscope at 400x magnification was used to assess immunohistochemical staining. H2A.X was scored as no expression (-), expression in 1–25% of cells (+), 26–50% of cells (++), 51–75% of cells (+++) and 76–100% of cells (++++) in the center, margin (between the center and periphery) or periphery. The intensity of the staining in each location was scored as 1 (low), 2 (moderate), 3 (high) or 4 (very high). The number of plus signs was added to the intensity number to obtain a weighted measure of the staining (italicized number). The assessment was based on observation of 6–40 spheroids for each experimental condition. Scale bar = 100 μM.

DSBs were clearly induced in spheroids by 6 Gy of radiation, as judged by the IHC assessment of gamma-H2AX expression (Figure 3). Although 100 μM 5-FU induced gamma-H2AX expression, the expression pattern after exposure to 5-FU + radiation was similar to that after radiation alone. However, the combination produced a higher intensity in the periphery. VLX600 at 1.5 and 3 μM clearly induced gamma-H2AX expression compared to control and the expression of gamma-H2AX after exposure to VLX600 + radiation was higher than after each exposure alone, with 3 μM VLX600 + 6 Gy increasing expression of gamma-H2AX in both the center and margin of spheroids.

## DISCUSSION

The lack of clinically relevant *in vitro* models in early drug screening may at least partly be responsible for the difficulties to identify and develop potent radiosensitizers for clinical use. One way that has been proposed to improve translation from preclinical results into the clinic is the introduction of three-dimensional tumor models in early drug screening [20]. Cell lines grown as spheroids are thought to more closely mimic solid tumors *in vivo* with respect to drug penetration, hypoxia/necrosis, metabolism, stem cell characteristics, proliferation, cell interaction and gene expression compared to monolayer cultures of human cell lines [16, 21].

Thus, the use of spheroid models is reasonably a way to better reflect the clinical situation when studying radiosensitizers. Historically, spheroid models have often been slow, technically demanding and not suitable for screening purposes [21]. Therefore, simplified high-throughput preclinical models that can identify synergistic effects between drugs and radiation at an early stage would be of substantial value.

The use of a clinically relevant outcome measurement is important when screening for new radiosensitizers. In this study, we used the FMCA and the GFP assay as outcome measurements of total cell kill and the results were then further explored in the ‘golden standard’ clonogenic assay. The qualitatively similar results retrieved for our hit VLX600 with both outcome measurements (Tables 1–3, Figure 1B–1D) argue for the use of total cell kill as a simpler read out. However, in spheroid experiments 5-FU exhibited radiosensitizing properties only in the clonogenic assay (Tables 1–3, Figure 1B and 1D).Therefore, total cell kill assays might miss marginally active drugs but could be used in high-throughput screening (HTS) experiments to sort out the most promising radiosensitizers for further evaluation.

GFP and FMCA readouts in total cell kill assays in spheroids were qualitatively similar (Tables 1–2) but the GFP assay is both faster and less laborious and simultaneously allows kinetic evaluation throughout the experiment.

In this study, the interaction between drug and radiation was analyzed in accordance with independent Bliss interaction, which assumes that the effect of a treatment is independent of the presence of the other treatment [22]. As a result, non-independent interactions between the two treatments would be seen as deviation from additivity rather than synergy. Therefore, the requirement to qualify for synergy in this study was high.

The reversal of hypoxic areas in tumors has been proposed as a promising strategy to overcome radiation resistance [11]. Since VLX600 has been shown to decrease oxygen consumption and reduce the hypoxic fraction of spheroids through inhibition of oxidative phosphorylation [16, 17], it is reasonable to believe that one of the important ways in which VLX600 exerts its radiosensitization effect is through reduced tumor hypoxia. This hypothesis is supported by the selective VLX600 radiosensitization in tumor spheroids compared to cells grown as monolayer, an effect that could not be seen with the other drugs used, including standard drugs and known radiosensitizers (Tables 1–2, and 4). The hypothesis is also strengthened by the increase in expression of gamma-H2AX in the hypoxic fraction of spheroids after exposure to the combination 3 μM VLX600 + 6 Gy compared to either treatment alone (Figure 3).

Proof of principle for sensitizing cancer cells to radiation through reducing the tumor hypoxic fraction was demonstrated decades ago in spheroids and xenografts using different mitochondrial poisons, although none of those were attractive to pursue in the clinic [23]. More recently, the clinically available drug arsenic trioxide (ATO) was shown to inhibit mitochondrial respiration, decrease oxygen consumption rate (OCR) of tumor cells and radiosensitize solid tumors in mice [23, 24]. However, although ATO is an established treatment for acute promyelocytic leukemia (APL), it has shown less efficacy in clinical trials in solid tumors and it has dose-related risks of cardiac and hepatic toxicity [25, 26].

Furthermore, the primary effect of the anti-diabetic drug metformin has been proposed to be inhibition of the mitochondrial electron transport chain and metformin has been shown to improve both tumor oxygenation and radiotherapy response [23, 27]. Moreover, the decrease in OCR in tumors through inhibition of mitochondrial complex III was recently shown to be associated with decreased tumor hypoxia and increased radiosensitivity [19]. Darinaparsin is another compound that has recently shown promising preclinical effects as a radiosensitizer of hypoxic cells [25]. However, preclinical and clinical data at this point are not strong enough to justify clinical use of the above drugs.

The identification of VLX600 as a radiosensitizer is promising since it has recently been shown to selectively act on cancer cells *in vitro* and also found to be active *in vivo* [16] and it is now in phase I clinical development in solid tumors (ClinicalTrials.gov Identifier: NCT02222363).

In conclusion, a new high-throughput compatible spheroid tumor model was used to study the interaction between drugs and radiation. The total cell kill assay in spheroids is suggested to be a feasible method in the search for novel radiosensitizers. Selective enhancement of radiation sensitivity in cells grown as tumor spheroids compared to cells grown as monolayer were seen with the novel iron-chelating inhibitor of oxidative phosphorylation VLX600, which makes it interesting in the development of a novel radiosensitizer.

## MATERIALS AND METHODS

### Cell lines and cell culture

The human colon cancer cell line HCT116 GFP (HCT116 cells transfected with Green Fluorescent Protein; Anticancer, Inc., San Diego, CA, USA) was cultured as detailed in Supplementary Materials and Methods.

### Drugs, irradiation, and cell culture experiments

#### Drugs

The drugs used in the experiments were the standard cytotoxic drugs 5-FU and oxaliplatin that are used as standard therapy for colorectal cancers (CRC) and are in clinical use also as radiosensitizers [3, 12, 28, 29]. Drugs previously considered to have a potential role as radiation sensitizers, *i.e.,* deferoxamine, ciclopirox, salinomycin, and tirapazamine, as well as the experimental drugs VLX50, VLX60 and VLX600, were also evaluated. For drug details, drug preparation and drug addition, see Supplementary Materials and Methods.

#### Spheroid experiments

The formation of HCT 116 GFP spheroids was recently described in detail [30]. Briefly, on day 0, 50 μl cell suspension with 10,000 cells were seeded into each well of a 384-well Corning^®^ black clear bottom ultra-low attachment (ULA) microplate (Corning Inc., New York, NY, USA). For further details about spheroid formation, see Supplementary Materials and Methods. Drug was added with the Echo 550 liquid handler (Labcyte) on day 7. Spheroids were then incubated with drug for 4–6 h before irradiation as described below for monolayer experiments. Following 7 days of drug incubation without change of culture medium cell viability was assessed in the fluorometric microculture cytotoxicity assay (FMCA), green fluorescent protein (GFP) assay and clonogenic assays, as described below.

#### Monolayer experiments

On day 0 of the experiment, 50 μl cell suspension (1,000 cells/well) was added into 384-well plates and allowed to pre-incubate overnight. On day 1, drug was added using the liquid handling system ECHO^®^ 550 (Labcyte Inc., Sunnyvale, CA, USA) and the plates were irradiated with an external low dose-rate gamma radiation source (GammaCell 40 Exactor, Best Theratronics, Canada) 4–6 h thereafter. Following 7 days of drug incubation without change of culture medium, cell viability was assessed in the FMCA, GFP- and clonogenic assays, as described below.

### Measurement of cellular cytotoxicity

#### Total cell kill assay

Cell kill in the total cell population was assessed using the FMCA and the GFP assay. The FMCA is based on conversion of fluorescein diacetate (FDA) to fluorescent fluorescein by viable cells with intact plasma membrane and has previously been described in detail [31].

Following spheroid experiments as described above, culture medium was removed and the spheroids dissociated into single cells by addition of 50 μl/well of Accumax (PAA, Pasching, Austria) and the plates were then incubated at 37°C for 30 min. Spheroids were then dissociated with a multipipette and the FMCA procedure was as described for the monolayer cultured cells (see below).

In the GFP assay for spheroids, after drug addition, the fluorescent signal generated from HCT116 GFP cells was measured every 24 h in the Cellomics ArrayScan VTI HCS Reader (Thermo Fisher Scientific). The Arrayscan software algorithm MEAN_ObjectAvgInten was used as the measure of mean spheroid fluorescence [30] and further used in the calculation of the AUTO SI defined as the spheroid fluorescence in experimental wells in percent of that in the same wells immediately before addition of drug 7 days earlier.

Following monolayer experiments as described above, culture medium was removed and after one wash in phosphate buffered saline (PBS), FDA buffer and FDA solution was added. After an incubation time of 50–70 min at 37°C, the fluorescence generated from each well was measured in the scanning fluorometer FLUOstar Optima (BMG Labtech, Ortenberg, Germany). Cell viability data is presented as survival index (SI) defined as the fluorescence in experimental wells in percent of that in unexposed control wells, with fluourescence of blank wells subtracted.

For the GFP assay in monolayers the fluorescence signal generated from HCT116 GFP cells was measured in the IncuCyte^®^ ZOOM Live-Cell Analysis System. One image was acquired in each well at one point and the IncuCyte^®^ software algorithm Green Object Confluence was used as the measure of fluorescence in each well and further used in the calculation of the SI, as defined above.

#### Clonogenic assay on spheroid cultures

Approximately 20 h after radiation, spheroids were dissociated into single cells as described above. The plates were then centrifuged, followed by removal of the Accumax solution and addition of 50 μl fresh medium. After mixing, 20 μl cell solution from each well was transferred together with 3 ml fresh medium to 6-well plates (Nunc) and the procedure was then identical to that described for monolayer experiments (see below).

#### Clonogenic assay on monolayer cultures

Approximately 20 h after radiation, 40 μl medium was removed from each well in the 384-well plates, followed by addition of 50 μl Accumax/well. After an incubation time of 10 min at 37°C, followed by mixing, the plates were centrifuged, Accumax solution removed and 50 μl fresh medium added. After mixing, 20 μl cell solution from each well was transferred to a tube with 3 ml fresh medium and the cell solution was, after mixing, directly transferred into each well in a 6-well plate (Nunc).

The 6-well plates were incubated at 37°C for 10 days. Cells were then fixed and stained as previously described by Franken *et al.* [32]. Briefly, after removal of medium and one wash in PBS, a mixture of 6% glutaraldehyde (Sigma-Aldrich) and 0.5% crystal violet (Sigma-Aldrich) was added to each well. After 30 min, the plates were rinsed with tap water and left to dry at air temperature. The plates were then photographed with a Canon iR-ADV C5235i printer and colonies counted on the computer screen. Cell survival data is presented as survival fraction (SF) defined as number of colonies in percent of that in unexposed control wells. This is a slightly modified SF definition since plating efficiency was not assessed.

#### Assessment of DNA double-strand breaks by immunohistochemistry

The spheroids were established and exposed to drugs and radiation as described above, 24 h after exposure to radiation, spheroids were harvested, washed in PBS, embedded in paraffin, sectioned and stained with the antibody Anti-gamma H2A.X (phospho S139 antibody [9F3] against the synthetic peptide phosphorylated (Ser139) human Histone H2A.X; ab26350; abcam, Cambridge) according to standard protocols. For further details, see Supplementary Materials and Methods.

### Data analysis and presentation

GraphPad Prism 7 was used for result calculations and graphical presentation. Results from concentration/dose - response curves are presented as means ± SEM for the number of experiments indicated. To characterize the interaction between drug and radiation, the mean SI (or SF) for wells treated with drug only (SI_d_ or SF_d_), and the mean SI (or SF) for wells irradiated only (SI_r_ or SF_r_) were used to calculate an expected combination SI (or SF) (SI_e_ or SF_e_) as follows: SI_d_ × SI_r_ = SI_e_ (or SF_d_ × SF_r_ = SF_e_) in accordance with independent Bliss interaction [22]. The SI (or SF) actually observed for the combination (SI_o_ or SF_o_) was then divided by SI_e_ (or SF_e_) to get an interaction ratio for each individual experiment. Tabular interaction data are presented as the mean interaction ratio (SI_o_/SI_e_ or SF_o_/SF_e_) for the number of experiments indicated and the one-sample *t*-test was used to calculate interaction ratios different from 1. A SI_o_/SI_e_ (or SF_o_/SF_e_) ratio <1 is considered to indicate synergy. Only drug concentrations with a SI_d_ or SF_d_ >25% are included in tabular interaction data. A two-tailed *p*-value of <0.05 was used to indicate interaction ratios significantly different from 1.

## SUPPLEMENTARY MATERIALS


