# Supplementary material for: A novel tumor spheroid model identifies selective enhancement of radiation by an inhibitor of oxidative phosphorylation
Source: Oncotarget. 2019 Sep 3;10(51):5372–82. doi: 10.18632/oncotarget.27166 (PMC6731106; doi:10.18632/oncotarget.27166)
Supplement: Supplementary file 1 [file oncotarget-10-5372-s001.pdf]

A novel tumor spheroid model identifies selective enhancement of radiation by an inhibitor of oxidative phosphorylation

SUPPLEMENTARY MATERIALS

MATERIALS AND METHODS

Cell lines and cell culture

The human colon cancer cell line HCT116 GFP (HCT116 cells transfected with Green Fluorescent Protein; Anticancer, Inc., San Diego, CA, USA) was cultured at 37°C in a humidified incubator containing 5% CO_2 in McCoy´s 5A medium supplemented with 10% heat-inactivated fetal bovine serum, L-glutamine (2 mM), penicillin (100 U/ml) and streptomycin (100 µg/ml).

The cell line was split twice a week and morphology and growth of cells were monitored on a weekly basis. Under these conditions, the doubling time for HCT116 GFP was (~18–20 h) as evaluated by confluence (phase contrast) and object confluence (green fluorescence) in a microscope that resides in the cell incubator (IncuCyte® ZOOM Live-Cell Analysis System; Essen BioScience, Ann Arbor, MI, USA). The cell line was authenticated by short-term repeat analysis performed by the cell bank. Morphology and growth of cells were monitored on a weekly basis. Cells were passaged for less than 6 months after resuscitation.

Drugs, irradiation and cell culture experiments

Drugs

Deferoxamine is an iron chelating radiosensitizing drug [1]. Ciclopirox is an antifungal compound and a cell membrane permeable iron chelator [2]. Salinomycin is an antibacterial drug that induces apoptosis in human cancer cells and has also radiosensitizing properties [3, 4]. Tirapazamine has selective toxicity for hypoxic cells in tumors and has demonstrated synergy in combination with radiation [5, 6].

The thiosemicarbazone drug VLX50 is an iron chelator and VLX60 is a copper chelate of VLX50 that has been shown to increase oxidative stress in tumor cells. Both drugs are in preclinical development [7]. VLX600 is in phase 1 clinical development for solid tumors and has recently been described as an iron-chelating inhibitor of oxidative phosphorylation with the ability to reverse hypoxia in spheroids [8, 9]. These drugs have mechanistic features making them potentially suitable as radiosensitizers and have been extensively studied in our laboratory and where therefore included in the study.

Source plates were prepared with appropriate concentrations of drugs in dimethyl sulfoxide (DMSO) and stored in the oxygen and moisture free MiniPod™ system (Roylan Developments Ltd, Surrey, UK) until drug was added to experimental plates using the liquid handling system ECHO® 550 (Labcyte Inc., Sunnyvale, CA, USA).

Spheroid formation

On day 0, 50 µl cell suspension with 10,000 cells were seeded into each well of a 384-well Corning® black clear bottom ultra-low attachment (ULA) microplate (Corning Inc., New York, NY, USA). The plates were placed on a Vari-Mix™ Platform Rocker (Thermo Fisher Scientific Inc., Waltham, MA, USA) in an angled position as described previously (see main manuscript) and incubated at 37°C in 5% CO_2. The slow movement of the rocker for 15 min every 3rd h allows for formation of homogenous and equally sized spheroids. MicroClime® lids (Labcyte Inc, Sunnyvale, CA, USA) were used to eliminate edge effects and protect against hydration or evaporation. On day 4, plates were put in a fixed second angled position which allows for movement of each spheroid to the lower left corner of each well. Drug was added with the Echo 550 liquid handler (Labcyte) on day 7.

Measurement of cellular cytotoxicity

Assessment of DNA double-strand breaks by immunohistochemistry (IHC)

The spheroids were established and exposed to drugs and radiation as described in Materials and Methods. Spheroids were harvested into Eppendorf tubes 24 h after exposure to radiation and the tubes were then centrifuged and washed once in PBS. Spheroids were embedded in paraffin and sectioned according to standard protocols. The antibody Anti-gamma H2A.X (phospho S139 antibody [9F3] against the synthetic peptide phosphorylated (Ser139) human Histone H2A.X; ab26350; abcam, Cambridge) was used to assess DNA double-strand breaks (DSBs). The commercially available MACH 1 Universal HRP-Polymer Detection kit (without protein block) was used in the staining process. Concentration and incubation time for the primary antibody was 1 µg/ml and 20 min respectively. A light microscope at 400x magnification was used to assess immunohistochemical staining.

REFERENCES

1. Cook J. Radiation sensitization of mammalian cells by metal chelators. Radiat Res. 2001; 155:304–310. https://doi.org/10.1667/0033-7587(2001)155[0304:rsomcb]2.0.co;2. [PubMed]
2. Kwong WL, Lok CN, Tse CW, Wong EL, Che CM. Anti-cancer iron(II) complexes of pentadentate N-donor ligands: cytotoxicity, transcriptomics analyses, and mechanisms of action. Chemistry. 2015; 21:3062–3072. https://doi.org/10.1002/chem.201404749. [PubMed]
3. Zhang Y, Zuo Y, Guan Z, Lu W, Xu Z, Zhang H, Yang Y, Yang M, Zhu H, Chen X. Salinomycin radiosensitizes human nasopharyngeal carcinoma cell line CNE-2 to radiation. Tumour Biol. 2016; 37:305–311. https://doi.org/10.1007/s13277-015-3730-6. [PubMed]
4. Fuchs D, Heinold A, Opelz G, Daniel V, Naujokat C. Salinomycin induces apoptosis and overcomes apoptosis resistance in human cancer cells. Biochem Biophys Res Commun. 2009; 390:743–749. https://doi.org/10.1016/j.bbrc.2009.10.042. [PubMed]
5. Spalding AC, Lawrence TS. New and emerging radiosensitizers and radioprotectors. Cancer Invest. 2006; 24:444–456. https://doi.org/10.1080/07357900600705706. [PubMed]
6. McKeown SR, Friery OP, McIntyre IA, Hejmadi MV, Patterson LH, Hirst DG. Evidence for a therapeutic gain when AQ4N or tirapazamine is combined with radiation. Br J Cancer Suppl. 1996; 27:S39–42. [PubMed]
7. Gullbo J, Fryknas M, Rickardson L, Darcy P, Hagg M, Wickstrom M, Hassan S, Westman G, Brnjic S, Nygren P, Linder S, Larsson R. Phenotype-based drug screening in primary ovarian carcinoma cultures identifies intracellular iron depletion as a promising strategy for cancer treatment. Biochem Pharmacol. 2011; 82:139–147. https://doi.org/10.1016/j.bcp.2011.04.003. [PubMed]
8. Zhang X, Fryknas M, Hernlund E, Fayad W, De Milito A, Olofsson MH, Gogvadze V, Dang L, Pahlman S, Schughart LA, Rickardson L, D'Arcy P, Gullbo J, et al. Induction of mitochondrial dysfunction as a strategy for targeting tumour cells in metabolically compromised microenvironments. Nat Commun. 2014; 5:3295. https://doi.org/10.1038/ncomms4295. [PubMed]
9. Fryknas M, Zhang X, Bremberg U, Senkowski W, Olofsson MH, Brandt P, Persson I, D'Arcy P, Gullbo J, Nygren P, Schughart LK, Linder S, Larsson R. Iron chelators target both proliferating and quiescent cancer cells. Sci Rep. 2016; 6:38343. https://doi.org/10.1038/srep38343. [PubMed]
